# Scientific Creativity: Discovery and Invention as Combinatorial

**DOI:** 10.3389/fpsyg.2021.721104

**Published:** 2021-08-23

**Authors:** Dean Keith Simonton

**Affiliations:** Department of Psychology, University of California, Davis, Davis, CA, United States

**Keywords:** combinatorial creativity, sciences, arts, development, personality

## Abstract

Although scientific creativity has often been described as combinatorial, the description is usually insufficiently formulated to count as a precise scientific explanation. Therefore, the current article is devoted to elaborating a formalization defined by three combinatorial parameters: the initial probability *p*, the final utility *u*, and the scientist’s prior knowledge of that utility *v*. These parameters then lead logically to an 8-fold typology involving two forms of expertise, two irrational combinations, and four “blind” combinations. One of the latter provides the basis for the definition of personal creativity as *c*=(1−*p*)*u*(1−*v*), that is, the multiplicative product of originality, utility, and surprise. This three-criterion definition then has six critical implications. Those implications lead to a discussion of various combinatorial processes and procedures that include a treatment of the No Free Lunch Theorems regarding optimization algorithms as well as the creativity-maximizing phenomena of mind wandering and serendipity. The article closes with a discussion of how scientific creativity differs from artistic creativity. Besides the obvious contrasts in the ideas entering the combinatorial processes and procedures, scientific combinations, products, and communities strikingly differ from those typical of the arts. These differences also imply contrasts in developmental experiences and personality characteristics. In sum, the formal combinatorial analysis enhances our understanding of scientific creativity.

## Introduction

Eminent figures in the sciences have often reported that their creativity, whether entailing discovery or invention, ultimately entails a combinatorial process or procedure. For example, Albert Einstein said “combinatorial play seems to be the essential feature in productive thought” (Hadamard, 1945, p. 147). Likewise, Poincaré (1921) once observed how “ideas rose in crowds; I felt them collide until pairs interlocked … making a stable combination” (p. 387). Not surprisingly, the combinatorial nature of creativity shows up in the resulting products. For instance, Thagard (2012) analyzed 100 breakthrough discoveries and 100 historic inventions, showing that, without exception, each could be broken down into a combinatorial product. The specific modalities and other attributes might vary, but their combinatorial nature was undeniable. Even those discoveries that Boden (2004) would call “exploratory” or “transformational” rather than “combinatorial” still entail combinations of representations of various kinds (e.g., Einstein’s special theory of relativity).

Speaking more generally, psychologists as far back as James (1880) have argued that all forms of creativity are essentially combinatorial (Simonton, 2018a). A prime illustration is the theory of creativity put forward by Mednick (1962): “the creative thinking process as the forming of associative elements into new combinations which either meet specified requirements or are in some way useful. The more mutually remote the elements of the new combination, the more creative the process or solution” (p. 221). In line with this position, computer programs that most effectively simulate human creativity operate according to combinatorial principles (e.g., evolutionary algorithms; Simon, 2013; see also Thagard and Stewart, 2011).

Here, I hope to develop the combinatorial theory of creativity five ways. First, I formalize the various types of combinations. This task is necessary because (a) not all combinations, nor even most, are creative and (b) non-creative combinations may still lead to creative combinations. Second, I specify the mandatory connection between combinatorial creativity and what has been variably styled “trial and error,” “generate and test,” “illumination and verification,” “blind variation and selective retention (BVSR),” etc. Third, I unify under a single specification all processes and/or procedures that generate potentially creative combinations. Fourth, I work out the repercussions for distinct domains of creativity, focusing on the contrasts between the sciences and the arts. Fifth and last, I indicate how the former contrasts lead to implications regarding the developmental experiences and personality characteristics of scientific vs. artistic creators.

Because the above already entails a fairly ambitious endeavor, I impose two constraints on my treatment. One, I focus on what is going on within a single scientist’s head, ignoring what also happens during lab meetings and other forms of group-level “brainstorming” (viz. cognitive rather than social psychology). Two, I concentrate on problem solving, so that the interest largely concerns combinations that constitute solutions to problems regarding natural phenomena, such as identification (“What is it?”), explanation (“How does it work?”), prediction (“What happens if?”), and invention (“By what device can this be done?”). That said, much if not most scientific creativity of the highest order easily abides by these two constraints.

## Formalization

Given a particular problem, ideational or behavioral combinations are generated that represent potential solutions. These combinations can be described by three crucial parameters that then lead to an 8-fold typology of combinatorial outcomes. One of these outcomes then describes a distinctively creative combination, whether it be a discovery or invention.

### Three Combinatorial Parameters (*p*, *u*, and *v*)

At the instant that problem solving starts, combinations may be described by the following three parameters (omitting subscripts; *cf*. Simonton, 2013a): *p*=the combination’s *initial probability* or “response strength,” where 0≤*p*≤1 (e.g., not spontaneously generated during the initial session, to generated after a certain delay within the session, and to instantaneously generated first); *u*=the combination’s *final utility* as a solution when fixed in the product, where 0≤*u*≤1 (e.g., completely useless, to merely satisficing, and to satisfying all specified criteria); and *v*=the *prior knowledge* of the combination’s final utility before generation, where 0≤*v*≤1 (e.g., utterly ignorant to “educated guess” to “justified true belief” as established by either past experience or logical deduction). It must be emphasized that all three parameters are defined with respect to the scientist’s subjective experience during the problem-solving episode, ignoring later consensual assessments by the scientific community, such as occurs in peer review and citation decisions. It should be noted that these proportions or probabilities can be easily converted from different measurement scales, such as Likert or rankings, by simple linear transformations. Hence, the 0–1 scaling imposes no limitations to empirical or theoretical inquiries.

These three parameters can be used to specify an 8-fold combination typology emerges (cf. Simonton, 2016, 2018b). Although Tsao et al. (2019) have offered a far more mathematically sophisticated quasi-Bayesian elaboration of this typology; the simpler version suffices for our current purposes.

### 8-fold Combination Typology

The typology comes from looking what happens when the three parameters approach either 0 or 1 (where “→”=“nears value of”), thus suggesting eight pure types. These are shown in Table 1. The types can then be grouped into the following three categories: expertise-driven, irrational, and “blind.”

**Table 1:** Typology based on the three combinatorial parameters.

Initial probability	Final utility	Prior knowledge	Qualitative description
*p*→1	*u*→1	*v*→1	Explicit expertise (e.g., algorithmic solutions)
*p*→0	*u*→0	*v*→1	Implicit expertise (e.g., “ruled out of court”)
*p*→1	*u*→0	*v*→1	Irrational perseveration (e.g., “definition of insanity”)
*p*→0	*u*→1	*v*→1	Irrational suppression (e.g., extraneous bias)
*p*→1	*u*→1	*v*→0	Lucky guess, or “right for the wrong reason”
*p*→1	*u*→0	*v*→0	Problem solving (e.g., expectation violation)
*p*→0	*u*→0	*v*→0	Mind wandering or behavioral tinkering
*p*→0	*u*→1	*v*→0	Creative idea or response

#### Two Expertise-Driven Combinations

The first two combinatorial types share the key characteristic that the utility value is already well-known in advance (*v*→1). That necessarily implies that the combination can provide little new knowledge. In fact, the two types represent the main guises of expertise. In the case of scientific creativity, this expertise includes domain-relevant knowledge and skills. On the one hand, maximal *explicit* expertise means that a given combination has a probability of unity because it enjoys a utility of unity and because that the utility is already well-known in advance. Like knowing how to solve a particular differential equation. Explicit expertise tends to be algorithmic, meaning that a fixed procedure guarantees a solution to a particular well-defined problem. On the other hand, maximal *implicit* expertise concerns the advance knowledge that certain combinations just will not work. That *a priori* assessment of the utility then renders the combination’s probability of emission close to zero. For instance, the law of the conservation of energy is now so well-established that no scientist would dare incorporate an overt violation of that law into any of their combinatorial products. If otherwise, then perpetual motion machines would be back on the table; the United States Patent Office currently rejects outright any applications that make such claims.

#### Two Irrational Combinations

A fundamental principle is illustrated by the above cases: As prior knowledge of the combination’s utility increases, the initial probability of the combination should correspond more closely with that utility. Hence, when *v*→1, then *p*→1 if *u*→1 and *p*→0 if *u*→0. This principle essentially represents a formal definition of rational behavior. Any organism can only successfully adapt to its environment by abiding by this 2-fold principle. In contrast, to violate this principle is to exhibit irrational behavior. In the cases of irrational perseveration, a combination continues to enjoy a high initial probability despite the fact that the person already knows that it has a low utility. This combinatorial type is echoed in the saying often misattributed to Einstein: “The definition of insanity is doing the same thing over and over and expecting different results.” The second irrational combination reverses the values of the initial probability and final utility while retaining the high value for prior knowledge. Such irrational suppression can have multiple sources, but one common cause may be an extraneous bias that interferes with a person acknowledging that an idea or response might be useful. In either case, it is evident that neither type of irrationality would prove relevant to scientific creativity. In this sense, this analysis argues against the “mad-genius hypotheses” that psychopathology is positively associated with creative thought and behavior (Simonton, 2019a).

#### Four “Blind” Combinations

The final four types of combinations share just one attribute, namely they all ensue out of near or complete ignorance of the utility (i.e., *v*→0). As a direct consequence, new knowledge can be acquired, albeit the specific nature of this acquisition will depend on the circumstances. The four types in Table 1 are described as follows:

1. The combination features a very high initial probability as well as a very high final utility, yet the prior knowledge of that utility is very low. This case is exemplified by the “lucky guess” or being “right for the wrong reason.” For instance, someone might win at the roulette table by impulsively putting all of their money on their birth year, but that would remain a matter of pure luck. Moreover, in this example, the person would not learn anything besides the very transient fact that the lucky number was the winning number at that specific bet. In contrast, someone else might guess the combination of numbers that opens a safe and thereby gain more enduring knowledge (at least until Security changes the combination). Of course, hackers can systematically search through common passwords without any prior knowledge other than those passwords are too often used by naïve computer users (e.g., “password” has a high probability).
2. The combination has a very high probability even though the utility is very low, an unfortunate occurrence rendered possible by the low prior knowledge of that utility. That is what separates this event from irrational perseveration. Although this combination may have more than one source, in the sciences, the best examples are what Kuhn (1970) called “anomalies.” These are occasions in which a prediction from a well-established (“paradigmatic”) theory or law is contradicted by data or derivation. Such anomalies demonstrate the limits to the scientist’s supposed expertise. Instead of problem solving, these events stimulate *problem finding*, and thus can lead to creativity even if these events are not creative in themselves (Rostan, 1994).
3. Here the initial probabilities are very low, the utilities very low, and the prior knowledge of those utilities also very low. This situation can be represented by either cognitive mind wandering or behavioral tinkering. The former is illustrated by reveries or daydreaming, the latter by playing around at the piano or trying out new moves on the dance floor. Most of the resulting combinations are very transient simply because they are so useless. But every so often, something surprisingly useful results. This outcome can occur simply because the prior knowledge is so low that the combinations cannot undergo preselection for utility, such as happens in implicit expertise. The surprising discovery then produces an insight or “ah-hah” experience that reveals that the combination actually qualifies for the fourth and final type.
4. Like the preceding type, the combinations here have low initial probabilities as well as low prior knowledge of the utilities, yet those utilities are actually very high. Of the eight types of combinations, this one is by far the most important for understanding scientific creativity. Indeed, combinations with the parameters *p*→0, *u*→1, and *v*→0 lead directly to a formal definition of creativity, as follows next.

### Three-Criterion Creativity Definition

Let a combination’s *originality* be defined by the inverse of the initial probability, namely, 1–*p*. In a like fashion, let a combination’s *surprise* be defined by the inverse of the utility’s prior knowledge value, namely, 1–*v*, where the latter gauges the amount of new knowledge gained from the combination. The combination’s *personal creativity* is then defined by the joint product of originality, utility, and surprise, or in more formal terms.

c=(1–p)u(1–v)

This three-criterion definition closely parallels those put forward by the United States Patent Office as well as other creativity researchers (*cf*. Amabile, 1996; Boden, 2004; Simonton, 2012c). However, it represents a departure from those who argue for some version of the two-criterion “standard definition” (Runco and Jaeger, 2012). The latter retains the first two criteria but omits the third, implicitly claiming that the scientist’s prior knowledge of the combination’s utility is irrelevant. Even so, it has been shown that this omission is both logically and psychologically untenable (Simonton, 2018b; Tsao et al., 2019). Not only is the above definition essential to fully capture the nature of creative combinations, but also the 8-fold typology must collapse into a 4-fold typology, merging combinatorial types that do not belong together. Most strikingly, perhaps, without the prior knowledge value *v*, explicit expertise is indistinguishable from a lucky guess and creativity is no different than irrational suppression! In the last case, prior knowledge is required to explain why a highly useful combination nonetheless features a rather low initial probability. Ignorance is the only reasonable answer.

### Six Implications of the Formal Definition

The definition of the personal creativity assigned to a given combination has six critical implications that follow directly from the formal specification.

#### First Implication

*The personal creativity associated with a combination is a continuous variable* (0≤*c*≤1). Just as the three criteria range from 0 to 1 inclusively, so must their product. Indeed, given the rarity of combinations satisfying all three criteria to perfection, their multiplicative outcome will most often fall in a middling range at best. To illustrate, suppose that the combination’s initial probability is small but non-zero, such as *p*=0.2 (so as to appear eventually in the initial problem-solving session), the final utility is not maximized but reasonably “satisficing,” or *u*=0.8 (so that most but not all of the usefulness criteria are met), and the prior knowledge of that utility adopts the form of an educated guess or “hunch,” so that *v*=0.5 (meaning that past data or logic provides some guidance *via* implicit expertise). Their product then yields *c*=0.32, or about one third up the theoretical scale. That value still far surpasses expertise-driven combinations that exhibit no creativity whatsoever (e.g., if *p*→1, *u*→1, and *v*→1, then *c*→0; see Simonton, 2013b).

#### Second Implication

*Whenever the personal creativity associated with a particular combination is much smaller than unity* (*i.e*., *c*<< 1), *creativity of a solution may represent an infinitely varied mixture of values for originality, utility, and surprise*. Yet, the qualitative character of the creative solution will differ depending on which of the three criteria predominates dominates. For instance, in pure scientific research, combinations may load higher on originality or surprise (e.g., the discovery of a new phenomenon in astrophysics), whereas in applied research, the combinations may load much higher on utility (e.g., the invention of a new vaccine). In short, combinatorial creativity cannot represent a homogeneous phenomenon. Two solutions to a certain problem might be equally creative even if one is more useful and the other more surprising.

#### Third Implication

*Given multiplicative rather than additive integration of the three criteria, each criterion becomes necessary but not sufficient for a combination’s personal creativity*. In other words, if any factor equals zero, then their product equals zero. Thus, a perpetual motion machine must have zero creativity (*c*=0), no matter how ingenious the device might appear (e.g., *p*=0.1), because such a device cannot work (*u*=0), a reality of which the would-be inventor was necessarily ignorant (*v*=0) unless the inventor intended the device as a practical joke (*v*=1). Accordingly, the number of ways that a combination might fail is multiple. Indeed, as seen in Table 1, only one combination out of eight types represents maximal creativity.

#### Fourth Implication

*Whenever the final utility has some non-zero value* (*i.e*., *u*>0) *and the prior knowledge of that utility has some value less than perfect* (*i.e*., *v*<1), *then the personal creativity associated with a given combination will maximize* (*i.e*., *c*→1) *when the initial probability is zero* (*i.e*., *p*=0), meaning that the combination will not appear early in the problem-solving episode. This implication relates directly to four stages of preparation, incubation, illumination, and verification of Wallas (1926), which are based on the experiences of superlative scientists, such as Helmholtz. In this classic sequence, an incubation period separates preparation from illumination. Yet according to the formal definition, incubation is not required for a creative combination to appear. Even so, those combinations that do require such a delay will prove the most creative, the other two parameters held constant under the specified conditions. At the same time, the formal definition does not imply that personal creativity will increase the longer incubation lasts. Nor should it. After all, the transition from incubation to illumination – the emergence of a combinatorial insight – is contingent on sundry external stimuli (see “opportunistic assimilation” in Seifert et al., 1995) and internal associations (see “constrained stochasticity” in Simonton, 2003; Carruthers, 2020) that can bear no relevance to the merits of the outcome. Certainly Archimedes’ famous Eureka experience would not have produced a more creative solution to the gold crown problem had he delayed taking a bath a day or more.

#### Fifth Implication

*Given multiplicative rather than additive integration, creative solutions are necessarily far more rare than noncreative solutions*. The distribution of personal creativity across all generated combinations will be approximately described by an inverse-power function in which the modal level of creativity will be zero. In contrast, highly creative combinations will be located at the end of a long and thin upper tail (see lower right quadrants in Figures 1, 2 from a Monte Carlo simulation; Simonton, 2012a). This outcome departs substantially from what would hold were the three criteria combined using additive integration, in which case the Central Limit Theorem would predict that the distribution would more closely approximate the “normal” or “bell-shaped” curve. Middling level creativity would then be far more commonplace. In that case, highly creative combinations would be about as common as uncreative combinations. That outcome just seems implausible. Most of the time scientists rely on explicit expertise, which represents the lowest level of creativity – publishable perhaps, but not innovative at all.

A corollary of this fifth implication is especially significant, namely, any scientist who seeks to produce more creative ideas will necessarily have to risk producing proportionately more non-creative ideas. In brief, quality of output should correlate positively with quantity of output (Simonton, 2010). Furthermore, this quantity-quality association should hold both across scientist careers (i.e., cross-sectionally) and within scientist careers (i.e., longitudinally).

#### Sixth Implication

*Because the solution utilities for creative combinations are unknown or incompletely known in advance of the generation of the potential solutions* (*i.e*., *v*<< 1), *then those solutions must undergo a second step of directed evaluation or assessment to determine the degree to which they truly solve the problem*. These two steps have been variously styled: trial and error (Bain, 1977), illumination and verification (Wallas, 1926), generate and test (various Artificial Intelligence algorithms), conjecture and refutation (Popper, 1963), “spontaneous behavior” plus selection by consequences (Skinner, 1981; Epstein, 1991), and blind variation and selective retention (BVSR; Campbell, 1960). This second step may adopt two forms: (a) a direct test against the external world, such as a laboratory experiment, or (b) an indirect test against an internal representation of the external world, such as a Gedanken experiment (*cf*. Skinnerian vs. Popperian in Dennett, 1995). Naturally, these two forms may be paired, the “thought experiment” selecting the best option to be subjected to a direct test. If the latter test reveals that the combination features lower than expected utility, then the internal representation of external reality will have to undergo revision. This scenario constitutes another guise of problem finding mentioned earlier.

Even in highly inspired “Ah-ha!” moments, acceptable utility is by no means guaranteed. Hence, Wallas (1926) was justified in adding the verification stage after the illumination stage to accommodate what might be styled “Oh, shucks!” events – what turn out to be false inspirations. The requirement for the second step increases with the complexity of the utility criteria. Although psychologists who conduct experiments regarding insight or problem solving often expect simple solutions that either work or do not work (i.e., *u* assumes dichotomous 0–1 values), in real-world creativity, the utility function is far more complex. For example, a complete explanatory model of DNA’s molecular structure has to satisfy several distinct criteria coming from different specialties (Watson, 1968). That is why its discovery deserved a Nobel Prize.

## Combinatorial Processes and Procedures

One crucial question remains to be addressed: Where do the potential solutions originate in the first place? The response is straightforward: Whatever works! This emphatic assertion echoes a claim of Feyerabend (1975) in *Against Method* that “anything goes.” To appreciate this point, we must recognize that creativity researchers have proposed an impressive number of processes and procedures, any one of which is capable of generating creative combinations (Simonton, 2017). A compilation might include remote association, divergent thinking, cognitive disinhibition (defocused attention), primary (primordial) process (“regression in the service of the ego”), dreams, psychoactive drugs, organic brain disorders, synesthesia, intuition, overinclusive (allusive) cognition, mind wandering, analogy, conceptual reframing (frame shifting), broadening perspective, juggling induction and deduction, problem dissection, reversal, tinkering, play, heuristic and systematic searches, serendipity, Geneplore, Janusian, Homospatial, and Sep-Con Articulation thinking (e.g., Ness, 2013; Rothenberg, 2015). Even if we acknowledge the possibility that some of these processes and procedures overlap to a certain degree, the fact remains that all work some of the time, but none works all of the time – and it is difficult to tell beforehand which will work best for a given problem. That is why so many have been identified in the first place. That is also another reason why the prior knowledge of the combination’s utility is so low, for the scientist is not even assured that a given line of attack will succeed.

This last claim parallels the famous No Free Lunch Theorems in algorithmic problem solving (Wolpert and Macready, 1997; see also Nickles, 2003). Although these theorems are multiple, complex, and mathematical, they can be summarized by saying that “All optimization algorithms perform equally well when averaged over all possible problems” (Simon, 2013, p. 614). Yet none works best across all possible problems. Extended to scientific creativity, no single process or procedure can guarantee highly creative solutions to all problems. Stated differently, a universally applicable “scientific method” for generating creativity cannot exist. Even so, each and every potential generator shares one key characteristic: the capacity to generate low probability potential solutions with unknown or incompletely known utility values (i.e., *p*→0 and *v*→0 while 0≤*u*≤1). The latter ignorance then requires a utility evaluation or test which assesses not just the specific combination but also the specific generator of the combination. If a generator consistently fails to produce a useful combination, then a different generator must be implemented, if a given scientist is able to do so.

Two phenomena illustrate the exceptional circumstances under which highly creative ideas are often generated in the sciences: one circumstance is internal (from inside the mind) and the other external (from the outside environment).

### Internal Circumstance: Mind Wandering

Boden (2004) observed that “*The bath*, *the bed*, *and the bus*: this trio summarizes what creative people have told us about how they came by their ideas” (p. 25, italics in original). That is, a creative combination can be spontaneously generated when engaged in some mundane or semi-alert activity, like taking a bath, waking up in bed, or boarding a bus. Recent research would connect this observation with the phenomenon of mind wandering (Gable et al., 2019). At the neuroscientific level, this phenomenon may entail the Default Mode Network in which the brain momentarily ceases to process external stimuli (Kühn et al., 2014). The main point is that this circumstance is ideal for generating low probability combinations with corresponding low prior knowledge values of the utilities. Because no selection or elicitation is possible based on those utilities, they can assume any value whatsoever (i.e., because *p*→0 and *v*→0, then 0≤*u*≤1). Although most of the time the combinations resulting from such reveries or daydreams will prove useless, every so often a highly useful combination can emerge, producing a “Eureka!” experience. The low likelihood of the last event is offset by the reality that people spontaneously lapse into mind wandering on numerous occasions throughout the day. That high frequency compensates the low base rates for useful ideas.

### External Circumstance: Serendipity

It has been observed often that discoveries and inventions can depend on some crucial chance stimulus or accidental event (Mach, 1896; Cannon, 1940). Well-known examples include penicillin, electromagnetism, X-rays, ozone, and the phonograph. Moreover, these events are by no means rare. In Thagard (2012) analyses of scientific discoveries about 25% relied on chance in some significant way. As a consequence, chance can play a breakthrough role in the history of science (Kantorovich and Ne’eman, 1989). This phenomenon is frequently termed “serendipity” (Roberts, 1989; Copeland, 2019). From the standpoint of the current combinatorial formalism, major serendipitous discoveries and inventions possess the unique parameter values: *p*=*v*=0 exactly, but *u*→1. In words, a highly useful combination could neither be deliberately generated nor its utility anticipated based on prior knowledge. For instance, Alexander Fleming discovered “bacteria-killing mold” when some staphylococci cultures were inadvertently exposed during his absence on a family vacation. Upon returning, he observed that a fungus that had contaminated one culture and was destroying the surrounding bacteria by apparently emitting some toxin. Fleming would never have thought of conducting an experiment in which he deliberately introduced such a contaminant (so *p*=0) and he would have no reason for doing so in the first place because molds were not then known to have antibacterial properties (so *v*=0).

The distinction is sometimes made between true serendipity, such as Fleming’s discovery of penicillin, and pseudo-serendipity, such as when Archimedes solved his gold crown problem. The former combination was not sought after, unlike the latter. Yet from the current analysis, this distinction has little importance. In both cases, *p*=*v*=0. The principal difference resides in the nature of the utility criterion. In pseudo-serendipity, the scientist or inventor already has a pretty good idea about what would constitute a useful combination. Indeed, the creator begins the problem-solving episode with those utility criteria in mind. In true serendipity, however, the utility of the discovery is recognized after the fact, and its full utility may not be immediately obvious. While working on a radio reception antenna for Bell Telephone Laboratories, Karl Guthe Jansky discovered that some radio waves originated in the center of the Milky Way galaxy. Even though he came to appreciate the potential value of this discovery, and wanted to pursue it further, his superiors saw any such effort as tangential to the development of trans-Atlantic communication systems, leaving the creation of radio astronomy to others.

Although mind wandering and serendipity illustrate means by which scientific creativity can become maximized, few discoveries and inventions attain the highest level of creativity. That point becomes more evident in the contrast between scientific and artistic creativity.

## Scientific Vs. Artistic Creativity

Although the primary focus of this article is to elaborate a combinatorial theory of scientific creativity, it must be recognized that artistic creativity is certainly combinatorial as well (Simonton, 2017). More specifically, combinatorial processes and procedures are important not just in the mathematical, physical, biological, and behavioral sciences, but also have a major part in the creativity exhibited in the visual arts, literature, and music. The latter assertion becomes evident from multiple sources, such as introspective reports, theoretical models, computer simulations, and single-case studies (Simonton, 2017). For instance, Pablo Picasso’s *Guernica*, arguably his most famous painting, is clearly a combinatorial product of various images and themes often found in his earlier works, such as the etching *Minotaurarchy* (Weisberg, 2004; Damian and Simonton, 2011). Yet if both scientific and artistic creativity are essentially combinatorial, how do they differ? If they do not differ, then there is no justification for concentrating this article on the former.

Naturally, one basic contrast is that the sciences represent distinct domains relative to the arts (Simonton, 2017). That means that creativity must be domain-specific to the extent that the ideas that are combined originate in different domains. The ideas that Einstein combined to produce his theories of relativity were utterly distinct from those that Picasso combined to create his *Guernica*. The former could not have yielded a painting about the horrors of war, nor could the latter have generated a journal article in theoretical physics.

Yet, another critical contrast between the arts and sciences concerns the degree to which the combinations are open to extra-domain ideas. Unlike what most often holds for creativity in almost all scientific domains, creativity in most artistic domains frequently incorporates ideas from everyday life and other experiences that are not strictly domain specific. Thus, Picasso could paint anything he wanted, whereas Einstein would seldom have any occasion to insert ideas from outside physics into his theories. Even Einstein’s thought experiments involved highly abstract representations of the everyday world. As a teenager, he once imagined what it might be like to speed alongside a light beam, a hypothetical scenario that helped inspire his special relativity theory about a decade later. Yet this is not something that could not even be conceived by anyone who was unfamiliar with some basic physics, such as the nature of electromagnetic waves.

Going beyond the above, I would like to return to the formal definition of creativity to offer more precise distinctions between scientific and artistic creativity. These concern the distinctive nature of scientific combinations, scientific products, and scientific communities.

### Scientific Combinations

On average, scientific combinations differ from those in the arts according to all three parameters in the three-criterion definition. Most obviously, the utility *u* is more precisely delineated in the sciences. For example, both fact and logic must be respected, whereas in the arts something akin to “poetic license” is often permitted. The latter is most apparent in science fiction where some basic laws of physics are routinely violated. In addition, the prior knowledge of the utility, or *v*, is more often much higher in the sciences than in the arts. That is, scientific combinations are more often expertise-driven hunches rather than random whims. The creative scientist frequently has theory or data on which to form an *a priori* assessment that an experiment or derivation will prove successful. Finally, and relatedly, the combination’s initial probability *p* is more likely higher in the sciences than in the arts, in part because pure originality is less valued in the former relative to the latter. Indeed, the scientific analog of “shock art” probably does not exist. Too often when a scientific combination is too original it tends to get ignored even when it is later determined to have been “ahead of its time” (Stent, 1972). A classic case is Gregor Mendel’s discovery of his genetic laws, a conceptual combination that took more than a third of a century to become fully appreciated.

### Scientific Products

Again on average, scientific products contain far more highly uncreative combinations than do artistic products. To appreciate the nature of this assertion, it must be acknowledged that any given creative product, whether a journal article, painting, poem, or composition, contains a hierarchical packaging of combinations. By “hierarchical,” I mean an organization in which some combinations operate at a higher level and others at a lower level. For instance, a standard symphony in the classical repertoire typically consists of three or four movements, each structured in a distinct way – such as sonata-allegro, song, minuet-trio, and rondo forms – and containing thematic material subordinated to those forms. Analogously, a journal article in science will contain introduction, method, results, and discussion sections each broken down into subsections – like the method section consisting of sample, measures, and analyses. The significant point is that in scientific products, much of the contents consist of “boilerplate,” such as the expected references, sample descriptions, measure inventory and administration, statistical analyses, and so forth. These segments of the scientific product are totally expertise driven. They contain combinations that graduate students and post docs can put together without supervision.

In stark contrast, artistic products contain a much larger proportion of combinations that are highly creative in themselves. This difference is most strikingly evident in poetry, where it would be unheard of for a poet to assign a stanza or so to a graduate student or post doc. Even when a poem features a recurrent refrain, each repetition takes on new meaning in the context of what occurs before. Great creative products in the arts do not rely on any boilerplate. Picasso’s *Guernica* may have introduced some images adapted from *Minotaurarchy*, but each undergoes combinatorial transformations that renders them dissimilar (e.g., the female holding the light).

### Scientific Communities

Once more, on average, the scientific community tends to exhibit a stronger consensus regarding the originality, utility, and surprise associated with the combinations making up creative products. In contrast, the consensual assessments regarding the combinations making up artistic products tend to be far less homogeneous. It must be underlined that at this point, I am deliberately switching from a purely personal assessment of combinatorial creativity to a consensual assessment of the combination’s creativity from the standpoint of those besides the creator who are in the best position to make those judgments. When that switch is made, then it becomes imperative to address the degree of correspondence between the two. As already noted, scientific combinations tend to feature less originality, more utility, and less surprise than holds for artistic combinations, a difference that would automatically support a stronger consensus on combinatorial creativity. Furthermore, the tendency for scientific combinations to contain more domain-specific components would also facilitate more consensus relative to those artistic.

However, to be more precise, it is necessary to recognize that the degree of consensus varies within the sciences as well (Fanelli and Glänzel, 2013; Simonton, 2015). For example, with respect to such consensual assessments, physics>chemistry>biology> psychology>sociology (Simonton, 2004, 2015). In short, the “hard” sciences exhibit stronger consensus than the “soft” sciences. Evidence also suggests that although the humanistic disciplines exhibit lower consensus than do the social sciences, the consensus in the humanities tends to exceed that in the arts, such as poetry (Simonton, 2009). In the latter domains, the concordance between personal and consensual assessments can be so small as to result in an ample history of “neglected geniuses” who were overlooked in their own day – such as the poet Emily Dickinson.

## Development and Personality

As just seen, although both scientific and artistic creativity are inherently combinatorial, they are not combinatorial in the same way. To express the difference in the simplest terms, scientific creativity is more constrained than artistic creativity, and thus intrinsically less creative in an absolute sense (Simonton, 2003). On the plus side, the constraints mean that scientists, relative to artists, can attain a stronger domain consensus on the merits of creative contributions. This contrast between scientific and artistic combinatorial creativity suggests parallel differences between the sciences and the arts with respect to the development and personality of creators in those contrasting domains.

### Developmental Experiences

A large number of events and conditions occurring in childhood and adolescence have been found to contribute to the development of creative potential that is later actualized during adulthood (Simonton, 2021). Yet a significant proportion of these circumstances can be subsumed under a single category, namely, “diversifying experiences that help weaken the constraints imposed by conventional socialization” (Simonton, 2000, p. 153). These experiences entail “highly unusual and unexpected events or situations that push individuals outside the realm of ‘normality’” (Damian and Simonton, 2015, p. 625; italics removed). To illustrate, “highly creative individuals stem from unconventional backgrounds (e.g., cultural or religious minorities, sickly dispositions, early orphanhood, or financial trouble), had unconventional educational and training experiences (e.g., studies abroad, multiple mentors, voracious reading, and diverse hobbies), and had more conspicuous leanings toward psychopathology ([in childhood and adolescence]”; Damian and Simonton, 2014, p. 389). It should be clear that such experiences would likely increase an individual’s capacity for generating original and surprising combinations, albeit without necessarily improving the ability for producing useful combinations. As a consequence, diversifying experiences should prove especially crucial in the development of creative artists, and that is in fact the case (Damian and Simonton, 2014). As a striking example, 30% of the Nobel laureates in literature laureates “lost at least one parent through death or desertion or experienced the father’s bankruptcy or impoverishment” whereas the Nobel laureates in physics “seem to have remarkably uneventful lives” (Berry, 1981, p. 387). It is easy to imagine how diverse traumatic experiences in childhood and adolescence might contribute directly to a novel or poem, but it is hard to conceive how the same events would improve a theory or experiment in the physical sciences.

Naturally, this last point relates directly with the earlier contrast between scientific and artistic creativity regarding the degree to which the combinations and products are strictly domain specific. This same domain specificity can be seen in the differential impact of formal education (Simonton, 2021). On the one hand, scientific creativity is most likely to appear in those individuals who were excellent students in science majors and who attained advanced degrees at top-notch universities (e.g., Chambers, 1964; Zuckerman, 1977; Segal et al., 1980; Simonton, 1992). Rigorous training is especially conspicuous in the natural sciences, such as physics, chemistry, and biology. On the other hand, artistic creativity is most likely to emerge in those persons who demonstrated far less scholarly competence and who less frequently earned higher degrees in their domains (e.g., Raskin, 1936; Simonton, 1986). Indeed, eminent poets are least likely to obtain any kind of domain-specific training beyond just learning how to read and write.

### Personality Characteristics

It has become abundantly clear, *via* decades of research, that creative achievement is most consistently and strongly associated with Openness to Experience, one of the personality dimensions in the Five Factor Model (McCrae and Greenberg, 2014). For example, Grosul and Feist (2014) showed that Openness in researchers in the physical, biological, and social sciences correlated with total citations and *h*-index score, but not with total productivity, showing that their publications had to have impact. Openness to experience exhibits prominent behavioral associations that are linked to scientific creativity, such as broad interests and avocations. For instance, highly creative scientists, such as Nobel laureates, are highly likely to engage in artistic activities (Root-Bernstein et al., 2008) and even display some degree of polymathy (Root-Bernstein and Root-Bernstein, 2020). Nor are these extra-scientific engagements mere superfluous entertainments, for often they make direct contributions to a scientist’s creativity (Root-Bernstein et al., 2008). A historic illustration is how Galileo was able to identify the dark and light patterns on the moon as indicating the presence of mountains, an observation that other astronomers had completely overlooked. Yet because Galileo had considerable interest and training in the visual arts, and even taught at an art school, his experience with chiaroscuro gave him a unique advantage (Simonton, 2012b). Indeed, he quickly published his own drawings of the lunar features that made the inferred peaks and valleys quite obvious.

An intellectual aspect of Openness to Experience also deserves mention, namely, that this personality factor is positively associated with cognitive disinhibition (Carson, 2014). The latter concerns the inability to filter out extraneous or irrelevant information, whether external stimuli or internal associations. In general, cognitive inhibition is advantageous because it prevents a person from being bombarded by information that just interferes with adaptive living by overwhelming processing and decision making. Even so, the tendency to ignore such input can also undermine creativity. This negative repercussion is perhaps most conspicuous in serendipity where a combinatorial event has a low initial probability of generation and a low prior knowledge of the utility (i.e., strictly speaking, *p*=*v*=0). A case in point is Fleming’s discovery of “bacteria-killing-mold.” Most bacteriologists of his time, when finding that one of their cultures had been so ruined, would simply have thrown the petri dish into the autoclave without a second thought, thus filtering out a potential breakthrough. That is an expertise-driven response (i.e., *p*=*u*=*v*=1) that gives the scientist a sterilized dish for growing a new culture for continuing research.

Of course, from what was said earlier, cognitive disinhibition should play a far more important role in artistic creativity than in scientific creativity. After all, artistic products contain a higher concentration of truly creative combinations than holds for scientific products (e.g., poem vs. journal article), and single artistic combinations relative to single scientific combinations tend to have lower initial probabilities as well as lower prior knowledge of the utilities (e.g., a line of poetry vs. a specific scientific hypothesis). In short, both the products and the combinations that make up the products are more original and surprising in the arts than the sciences (even if the utilities might be more comparable given the distinct utility criteria). In line with these art-science contrasts, Openness to Experience is more conspicuous in artistic creators than in scientific creators (Feist, 1998). Yet there is also a downside to this differential, one relating to the associated cognitive disinhibition. The latter mental proclivity is not just associated with creativity but also with mental illness (Carson, 2014). And it is easy to see why: If too much unfiltered information is allowed into cognitive processing, then the latter may become overwhelmed, becoming increasingly unable to distinguish the real from the unreal. Accordingly, in comparison to scientific creators, artistic creators must more often walk a fine line between adaptive creativity and maladaptive psychopathology, a balance that is not always maintained. This psychological vulnerability is only accentuated by the fact that, as also noted earlier, consensus on what is creative and what not is appreciably higher in the sciences than in the arts, leaving the creative artist more isolated than the creative scientist.

Although the methodological issues are extremely complex (Simonton, 2019a), the available empirical data, whether psychometric or historiometric, supports the above theoretical expectation (e.g., Ludwig, 1992; Post, 1994; Simonton, 2014). In general, not only will creative scientists exhibit less psychopathology than creative artists, but also the degree of psychopathology will be negatively associated with scientific creativity while positively associated with artistic creativity. That said, this generalization must be rendered more nuanced because additional contrasts occur within scientific and artistic domains. For instance, Ludwig (1998) demonstrated that “Persons in professions that require more logical, objective, and formal forms of expression tend be more emotionally stable than those in professions that require more intuitive, subjective, and emotive forms” (p. 93). Thus, within literary creativity, nonfiction writers are more emotionally stable than fiction writers, who in turn are more stable than poets. And within the sciences, natural scientists are more emotionally stable than social scientists. Fitting Ludwig’s demonstration, Ko and Kim (2008) showed that psychopathology was negatively associated with the eminence of those scientists who sought to preserve the received paradigm, whereas for those scientists who challenged that paradigm the same association was positive. Presumably, the latter revolutionary scientists would incline toward more cognitive disinhibition in order come up with their unusually creative alternatives to traditional ideas.

Any treatment of the relation between creativity and psychopathology must take care to emphasize that we are usually speaking of subclinical symptoms rather than outright mental illness. It goes without saying that when an individual’s symptoms reach the highest levels, creativity ceases altogether. Generating combinations having the parameter values *p*=1, *u*=0, and *v*=1 is decidedly irrational, not creative. Plus, suicide or addiction terminates the career.

## Conclusion

This article elaborates a combinatorial analysis of scientific creativity. The elaboration is founded on a formal three-criterion definition of what constitutes a creative combination. This definition has six significant implications about discovery and invention, implications that also help distinguish between scientific and artistic creativity. The latter distinction also proves instructive in understanding how development and personality differ in the arts and sciences. These formal developments should improve understanding of scientific creativity on both theoretical and empirical grounds. In the former case, this treatment should contribute to future mathematical models and computer simulations; most to date operate without the benefit of the eightfold typology (e.g., Langley et al., 1987; Simonton, 1997; Thagard and Stewart, 2011; *cf*. Tsao et al., 2019). In the case of future empirical research, testable predictions can be derived from the implications of the formal creativity definition. For example, the fifth implication leads to the prediction that across a scientist’s entire career, high-impact, or “breakthrough” ideas should be relatively rare, the bulk of their output consisting of more mediocre contributions. This prediction has actually been confirmed in a recent empirical study that showed that this expectation even holds for Nobel laureates (Sinatra et al., 2016). Equally important is this study’s finding that high-impact was positively correlated with total output, supporting the corollary of this fifth implication (see also Simonton, 1997).

Nevertheless, the full repercussions of this combinatorial analysis in all likelihood go well beyond what could be presented in this relatively brief essay. Most notably, the current presentation focused on the individual scientist or inventor. This individualistic or “cognitivist” perspective is certainly appropriate in any psychology of creative science, which constitutes the topic of this journal section. Yet it also must be recognized that the scientific community plays a much bigger role than has been treated here (Simonton, 2019b). This involvement leads to creative phenomena that cannot be explicated in purely psychological terms. An obvious example is the phenomenon of multiple discovery and invention, that is, where two or more creators come up with the same idea independently. Even though these multiples can also be explained as combinatorial outcomes, that explanation requires that the analysis operate at the level of scientific and technological disciplines (Simonton, 2010). Therefore, a comprehensive understanding of creative science demands the incorporation of a sociological perspective – sociology of science coupled with a psychology of science.

## Data Availability Statement

The original contributions presented in the study are included in the article/supplementary material; further inquiries can be directed to the corresponding author.

## Author Contributions

The author confirms being the sole contributor of this work and has approved it for publication.

## Conflict of Interest

The author declares that the research was conducted in the absence of any commercial or financial relationships that could be construed as a potential conflict of interest.

## Publisher’s Note

All claims expressed in this article are solely those of the authors and do not necessarily represent those of their affiliated organizations, or those of the publisher, the editors and the reviewers. Any product that may be evaluated in this article, or claim that may be made by its manufacturer, is not guaranteed or endorsed by the publisher.
